# VAMP-2 is a surrogate cerebrospinal fluid marker of Alzheimer-related cognitive impairment in adults with Down syndrome

**DOI:** 10.1186/s13195-021-00861-0

**Published:** 2021-06-28

**Authors:** Alberto Lleó, Maria Carmona-Iragui, Laura Videla, Susana Fernández, Bessy Benejam, Jordi Pegueroles, Isabel Barroeta, Miren Altuna, Silvia Valldeneu, Mei-Fang Xiao, Desheng Xu, Raúl Núñez-Llaves, Marta Querol-Vilaseca, Sònia Sirisi, Alexandre Bejanin, M. Florencia Iulita, Jordi Clarimón, Rafael Blesa, Paul Worley, Daniel Alcolea, Juan Fortea, Olivia Belbin

**Affiliations:** 1 Memory Unit and Biomedical Research Institute Sant Pau (IIB Sant Pau), Neurology Department, Hospital de la Santa Creu i Sant Pau, Autonomous University of Barcelona, c/Sant Quintí, 77-79, 08025 Barcelona, Spain; 2 Centro de Investigación Biomédica en Red sobre Enfermedades Neurodegenerativas (CIBERNED), 28031 Madrid, Spain; 3 Barcelona Down Medical Center, Fundació Catalana Síndrome de Down, Barcelona, Spain; 4 Solomon H. Snyder Department of Neuroscience, Johns Hopkins University School of Medicine, Baltimore, MD 21205 USA; 5 Department of Neurology, Johns Hopkins University School of Medicine, Baltimore, MD 21205 USA

**Keywords:** Down syndrome, Alzheimer’s disease, Synapse, Biomarker, Cognitive decline

## Abstract

**Background:**

There is an urgent need for objective markers of Alzheimer’s disease (AD)-related cognitive impairment in people with Down syndrome (DS) to improve diagnosis, monitor disease progression, and assess response to disease-modifying therapies. Previously, GluA4 and neuronal pentraxin 2 (NPTX2) showed limited potential as cerebrospinal fluid (CSF) markers of cognitive impairment in adults with DS. Here, we compare the CSF profile of a panel of synaptic proteins (Calsyntenin-1, Neuroligin-2, Neurexin-2A, Neurexin-3A, Syntaxin-1B, Thy-1, VAMP-2) to that of NPTX2 and GluA4 in a large cohort of subjects with DS across the preclinical and clinical AD continuum and explore their correlation with cognitive impairment.

**Methods:**

We quantified the synaptic panel proteins by selected reaction monitoring in CSF from 20 non-trisomic cognitively normal controls (mean age 44) and 80 adults with DS grouped according to clinical AD diagnosis (asymptomatic, prodromal AD or AD dementia). We used regression analyses to determine CSF changes across the AD continuum and explored correlations with age, global cognitive performance (CAMCOG), episodic memory (modified cued-recall test; mCRT) and CSF biomarkers, CSF Aβ_42:40_ ratio, CSF Aβ_1-42_, CSF p-tau, and CSF NFL. P values were adjusted for multiple testing.

**Results:**

In adults with DS, VAMP-2 was the only synaptic protein to correlate with episodic memory (delayed recall *adj.p* = .04) and age (*adj.p* = .0008) and was the best correlate of CSF Aβ_42:40_ (*adj.p* = .0001), p-tau (*adj.p <* .0001), and NFL (*adj.p <* .0001). Compared to controls, mean VAMP-2 levels were lower in asymptomatic adults with DS only (*adj.p* = .02). CSF levels of Neurexin-3A, Thy-1, Neurexin-2A, Calysntenin-1, Neuroligin-2, GluA4, and Syntaxin-1B all strongly correlated with NPTX2 (*p* < .0001), which was the only synaptic protein to show reduced CSF levels in DS at all AD stages compared to controls (*adj.p* < .002).

**Conclusion:**

These data show proof-of-concept for CSF VAMP-2 as a potential marker of synapse degeneration that correlates with CSF AD and axonal degeneration markers and cognitive performance.

## Introduction

Alzheimer’s disease (AD) is the leading cause of death in adults with Down syndrome (DS), with a cumulative incidence that exceeds 90% in the seventh decade [1–4]. Current standard cerebrospinal fluid (CSF) markers for AD in the DS population are restricted to surrogate markers of amyloidosis (Aβ_42:40_ ratio, Aβ_1-42_) and tau-mediated neurodegeneration (p-tau) and neurofilament light chain combined with neuropsychological assessment. However, neuropsychological assessment can be confounded by substantial inter-individual variation in intellectual disability (ID). Therefore, there is an urgent need for objective markers of AD-related cognitive impairment in people with DS to improve diagnosis, monitor disease progression, and assess response to disease-modifying therapies.

Synapse loss is an early event in AD [5] and one of the best pathological correlates of cognitive dysfunction [6–9]. As such, synaptic proteins that show AD-associated changes in biofluids are rapidly gaining attention as potential surrogate markers of AD-related synapse loss and may be informative markers of early AD-related cognitive dysfunction in adults with DS.

Neuronal pentraxin-2 (NPTX2), a protein involved in inhibitory circuit dysfunction [10], is a promising biofluid surrogate marker of inhibitory circuit dysfunction and cognitive decline in sporadic AD [11–13], vascular dementia [14], genetic frontotemporal dementia [15], and Lewy body dementia [16]. We recently reported low CSF NPTX2 concentrations in adults with DS across the AD continuum, which correlated with cortical atrophy and reduced glucose metabolism. However, CSF NPTX2 levels did not correlate with measures of cognitive decline in our DS cohort [17]. In the same study, we also evaluated the glutamatergic receptor, GluA4, and found no association with cognitive measures.

The aim of this study was to evaluate a comprehensive panel of alternative synaptic proteins (Calsyntenin-1, Neuroligin-2, Neurexin-2A, Neurexin-3A, Syntaxin-1B, Thy-1, VAMP-2) as surrogate markers of early AD-related cognitive decline in non-trisomic cognitively normal controls (n = 20) and a large cohort of adults with DS (n = 80) from across the preclinical and clinical AD continuum, exploring their relationship to cognitive performance. The panel comprises 8 proteins that were shown to be specifically expressed at the synapse in human frontal cortex postmortem tissue and show CSF alterations that precede clinical symptoms and markers of neurodegeneration in sporadic AD [18]. We also compare the CSF profile of the synaptic panel proteins in adults with DS to that of previously published data on NPTX2 and GluA4 in the same cohort [17].

## Material and methods

### Objectives

The primary objective of this study was to evaluate a comprehensive panel of synaptic proteins as surrogate markers of early AD-related cognitive decline in adults with DS from across the preclinical and clinical AD continuum, specifically exploring their relationship to cognitive performance and AD biomarkers.

### Study design

This is a single-center, cross-sectional study of CSF levels of synaptic markers in adults with DS, sporadic AD patients and cognitively normal controls. The study (IIBSP-BMS-2018-103) was approved by the local ethics committee (Comité Ètic d’Investigació Clínica, Fundació de Gestió Sanitària de l’Hospital de la Santa Creu i Sant Pau) and was conducted in accordance with the Declaration of Helsinki. All participants gave their written informed consent to participate in the study. Non-trisomic controls were selected from the Sant Pau Initiative in Neurodegeneration (SPIN) cohort, a prospective longitudinal cohort at Hospital Sant Pau, Barcelona, Spain [19]. Adults with DS were selected from the Down Alzheimer Barcelona Neuroimaging Initiative (DABNI), a prospective longitudinal cohort, linked to a population-based health plan in Catalonia, Spain, led by the Fundació Catalana Síndrome de Down and Hospital de Sant Pau [20]. Inclusion criteria for controls required the absence of a cognitive or neurological disorders and normal CSF core AD biomarker (Aβ_1-42_, Aβ_42/40_ ratio, t-tau, p-tau) concentrations using our validated cut-offs for sporadic AD [21]. For adults with DS, inclusion criteria for participation in the study required that all participants were over 18 years of age. Where consent was given, participants received a comprehensive neurological and neuropsychological evaluation [22] and underwent a lumbar puncture to assess CSF biomarkers [20]. As in previous studies [4, 20], participants with DS were classified by neurologists and neuropsychologists, blind to biomarker data in a consensus meeting into asymptomatic AD (aDS), prodromal AD (pDS), and AD dementia (dDS) according to previously published criteria [20].

### Neuropsychological assessment

The level of ID in adults with DS was categorized according to the Diagnostic and Statistical Manual of Mental Disorders (DSM), Fifth Edition, as mild, moderate, severe, or profound ID, based on caregivers’ reports of the individuals’ best-ever level of functioning and the Kaufmann Brief Intelligence Test (KBIT) [23]. As previously described [20, 22], neurological and neuropsychological examination of the full range of cognitive impairment included a semi-structured health questionnaire (Cambridge Examination for Mental Disorders of Older People with Down Syndrome and others with intellectual disabilities [CAMDEX-DS]) [24] and a neuropsychological battery including the Cambridge Cognition Examination (CAMCOG) adapted for intellectual disabilities in DS participants and was restricted to those with mild and moderate ID. The Spanish version of the cued recall test modified for use in people with ID (mCRT) [25] was used to evaluate episodic memory as previously described [26]. The total mCRT scores for immediate recall were calculated as free recall score + cued recall score.

### CSF collection, biomarker assessment

CSF samples were collected following international consensus recommendations [27] as previously described [28]. Samples had been previously stored at − 80 °C and had not been thawed prior to analysis. Commercially available fully automated immunoassays were used to determine levels of CSF Aβ_1-42,_ Aβ_1-40_, NFL, total tau, and p-tau at threonine residue 181 (Lumipulse Aβ_1-42 ,_ Aβ_1-40_, total tau G, p-tau 181, Fujirebio-Europe, NFL Simoa Quanterix, MA, USA) [21].

### Targeted liquid chromatography mass spectrometry (LC-SRM)

We monitored a set of 22 proteotypic peptides corresponding to 10 proteins (Calsyntenin-1, GluA2, GluA4, Neurexin-2A, Neurexin-3A, Neuroligin-2, Syntaxin-1B, Tenascin-R, Thy-1 and VAMP-2) using the previously described selected reaction monitoring (SRM) method [18]. Briefly, we digested individual CSF samples overnight and spiked isotopically labeled peptides (Pepotech SRM custom peptides, grade 2, Thermo Fisher Scientific) into each sample. We analyzed an equivalent of 5 μl of each sample in a randomized order over a 120-min gradient (0–35% ACN + 0.1% FA) in SRM mode using a triple quadrupole-Qtrap mass spectrometer (5500 QTrap, Sciex, Masachussetts) coupled to a nano-LC chromatography column (300 μl/min, 25-cm C18 column, 75 μm I.d., 2 μm particle size). We ran BSA technical controls between each sample. We used isotopically labeled peptides as internal standards. We visualized and analyzed transitions using Skyline 3.5 as previously described [18]. To evaluate the stability of the peptides over the course of the experiment, we injected a pool of all the samples over the duration of the mass spectrometric measurements and monitored the peak area of the standard peptides. The GluA2 peptide was unstable and removed, thus resulting in the exclusion of GluA2 from the study. We processed the SRM transitions using the dataProcess function of MSstats v3.5 package in R [29] and removed transitions with between-run interference (betweenRunInterferenceScore< 0.8). One censored transition (VAMP-2 peptide) where endogenous log2 intensity was below the detection cut-off designated by the MSstats package (8.49) was removed. We used the EqualizeMedians function to normalize the transitions and Tukey’s Median Polish to generate a summarized value of transitions for each protein. Two peptides (Calsyntenin-1 and Neurexin-3A) were excluded from the summarization as the endogenous peptide was not detected in all samples. The results for the two Tenascin-R peptides are not reported here due to the lack of synapse specificity of Tenascin-R [18]. Data for the 3 GluA4 peptides from the same SRM experiment have been reported previously [17].

### Statistical analysis

All statistical analyses were performed in R version 3.4.3 [30]. We excluded 1 data-point each for Neurologin-2, Neurexin-3A, Syntaxin-1B, Thy-1, VAMP-2, NPTX2 p-tau, and t-tau as outliers due to violation of the 3 × interquartile range rule. The outlier values were from 3 different samples from the DS group. Group comparisons were performed using χ2 test, t test, or linear regression. Where regression residuals deviated from a Gaussian distribution (Shapiro-Wilk p < 0.05), tests were performed on square root or log2 transformed values, which did not deviate from a Gaussian distribution (Shapiro-Wilk p > 0.05). Raw values were used for those sub-analyses. We used Pearson coefficients to assess correlations. However, to account for the ceiling effect of cognitive tests we used Spearman coefficients on raw values to assess correlations with cognitive measures. Linear regressions of cognitive data were performed on raw data as transformations did not improve the distribution. When comparing the association of multiple synaptic proteins, p values were adjusted for multiple testing using the Benjamini-Hochberg method.

## Results

### Demographics

Table 1 shows the demographic and clinical data for the participants included in the study, which included 20 controls and 80 adults with DS from across the AD continuum (40 aDS, 19 pDS, and 21 dDS). The mean age-at-analysis across the whole study was 44.5 years (standard deviation; SD = 11.2). Compared to controls, the mean age was comparable in pDS (+ 5 years, *p* = .20) and dDS (+ 5 years, *p* = .13) but lower in aDS (− 12 years, *p* < .0001). The male to female proportion was comparable across clinical groups (*p* = .45). The level of ID in the adults with DS was classified as either mild/moderate (78% of cases) or severe/profound (22% of cases), a proportion that was comparable across clinical groups (*p* = .37). Cognitive tests were restricted to individuals with mild or moderate intellectual disability. As would be expected, cognitive scores were sequentially lower in pDS (CAMCOG; − 11, *p* = .02, mCRT immediate; − 15, *p <* .0001, mCRT delayed; − 5, *p <* .0001) and dDS (CAMCOG − 21 *p <* .0001, mCRT immediate; − 20. *p <* .0001, mCRT delayed; − 7, *p <* .0001) compared to aDS. As previously reported [20], the mean Aβ_42:40_ ratio (all *p* < .0001) was lower in all DS groups compared to controls, while mean CSF p-tau and t-tau levels were higher in pDS and dDS compared to controls (all *p* < .0001). CSF NFL levels were available for DS only and were elevated in pDS and dDS compared to aDS (*p* < .00001).

**Table 1:** Demographics and clinical data for study participants

	Controls	aDS	pDS	dDS
N	20	40	19	21
Age-at-analysis, years	**47** (11, 24–64)	**35** (9, 22–57)^b^	**52** (4, 45–60)	**52** (5, 42–62)
% Female	**60%**	**40%**	**42%**	**38%**
% Mild or moderate ID	**0%**	**83%**	**79%**	**67%**
CAMCOG score^a^	**NA**	**80/107** (11, 55–96, *n* = 31)	**70/107** (13.8, 41–92, *n* = 11)^c^	**59/107** (13.9, 39–87, *n* = 10)^c^
mCRT score (immediate)^a^	**NA**	**35/36** (1.5, 30–36, *n* = 30)	**20/36** (11.2, 0–36, *n* = 12)^c^	**15/36** (7.9, 0–32, *n* = 11)^c^
mCRT score (delayed)^a^	**NA**	**12/12** (0.9, 8–12, *n* = 31)	**6/12** (3.8, 0–12, *n* = 13)^c^	**4/12** (3.3, 0–12, *n* = 11)^c^
CSF Aβ_42:40_ ratio	**0.11** (0.01, 0.08–0.12)	**0.09** (0.02, 0.04–0.12)^b^	**0.05** (0.01, 0.03–0.08)^b^	**0.05** (0.01, 0.04–0.08)^b^
CSF p-tau pg/ml	**36** (8, 22–54)	**35** (24, 10–122)	**145** (86, 22–304)^b^	**158** (82, 31–323)^b^
CSF t-tau pg/ml	**243** (57, 167–366)	**295** (166, 86–671)	**936** (658, 118–2565) ^b^	**959** (500, 212–1988) ^b^
CSF NFL pg/ml	**NA**	**355** (234, 65–1036)	**1071** (767, 313–3123)	**1387** (832, 627–3957)

Mean values (standard deviation, range) are given for each variable across clinical groups. NA, not available. ^a^In individuals with mild/moderate intellectual disability (ID) only. ^b^p < 0.05 compared to controls. ^c^p < 0.05 compared to aDS

### CSF VAMP-2 levels show a distinct profile to other synaptic proteins in adults with DS

**Fig. 1:**
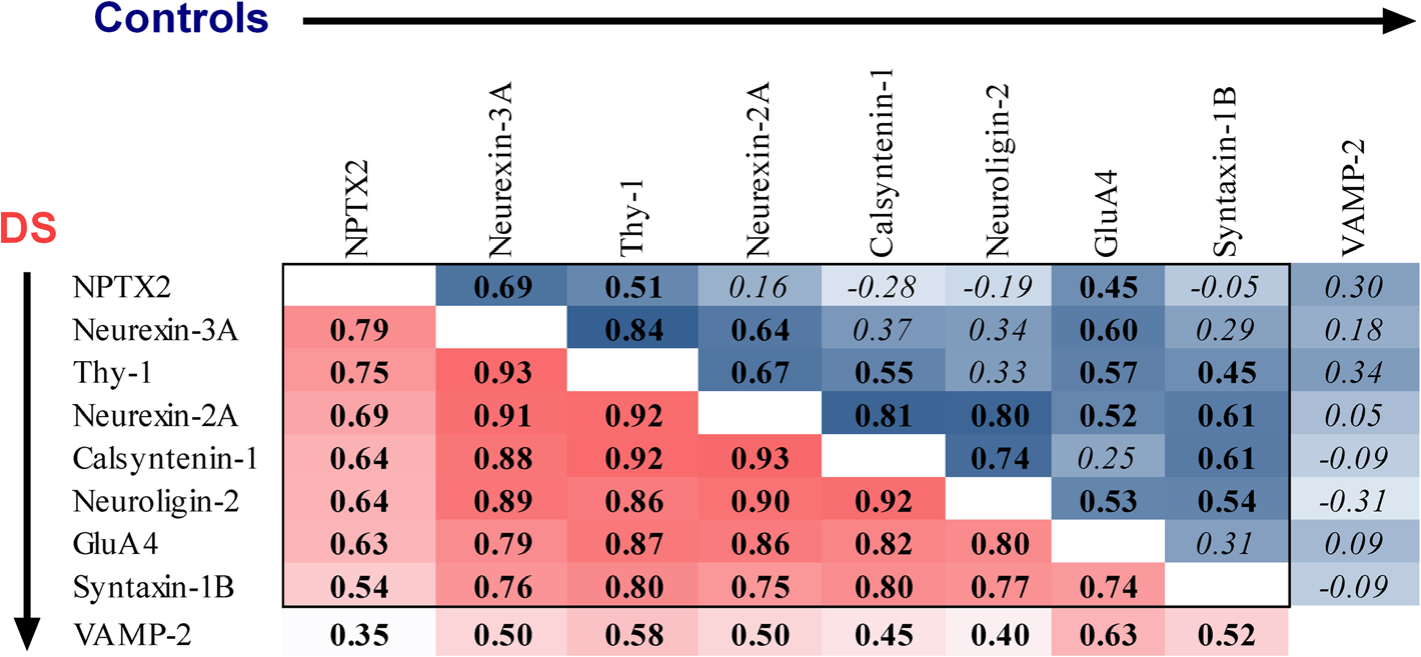
Pair-wise correlation coefficients of CSF levels of 9 synaptic proteins in DS and controls. r coefficients resulting from statistical tests performed in the DS group (red) and controls (blue) for the 8 synaptic panel proteins and NPTX2 are shown. Degree of shading is relative to size of r coefficients, which are shown in bold where p < .05 and italicized where p > .05. NPTX2 and GluA4 data for these samples are published in [17]

The synaptic panel analyzed here includes 7 synaptic proteins previously unpublished in this cohort (Calsyntenin-1, Neurexin-2A, Neurexin-3A, Neuroligin-2, Syntaxin-1B, Thy-1, and VAMP-2) and their comparison to 2 synaptic proteins, NPTX2 and GluA4, previously reported in the same cohort [17]. We first sought to determine the degree of correlation between CSF levels of the 9 synaptic proteins. Figure 1 shows that in adults with DS, synaptic proteins, including Neurexin-3A, Thy-1, Neurexin-2A, Calysntenin-1, Neuroligin-2, GluA4, and Syntaxin-1B, were all correlated (pair-wise *r* = .70 to .96, *n* = 78-80, *p* < .0001). They also all correlated with NPTX2 (pair-wise *r* = .56 to .84, *n* = 78-79, *p* < .0001). VAMP-2 showed the weakest correlation with all other proteins (*r* = .47 to.69, n = 78-80, *p* < .0001). In controls, all proteins showed weaker pair-wise correlations than in the DS group, although NPTX2, Neurexin-3A, Thy-1, Neurexin-2A, Calysntenin-1, Neuroligin-2, GluA4, and Syntaxin-1B were moderately correlated in at least one pair-wise combination (pair-wise *r* = .46 to .87, *n* = 20, *p* < .04). VAMP-2 did not correlate with any other protein in controls (pair-wise *r* = -.34 to .29, *n* = 20, *p* > .14). We took VAMP-2 forward for further analyses due to its relative independence from NPTX2.

### CSF VAMP2 changes over the course of AD and with age in adults with DS

**Fig. 2:**
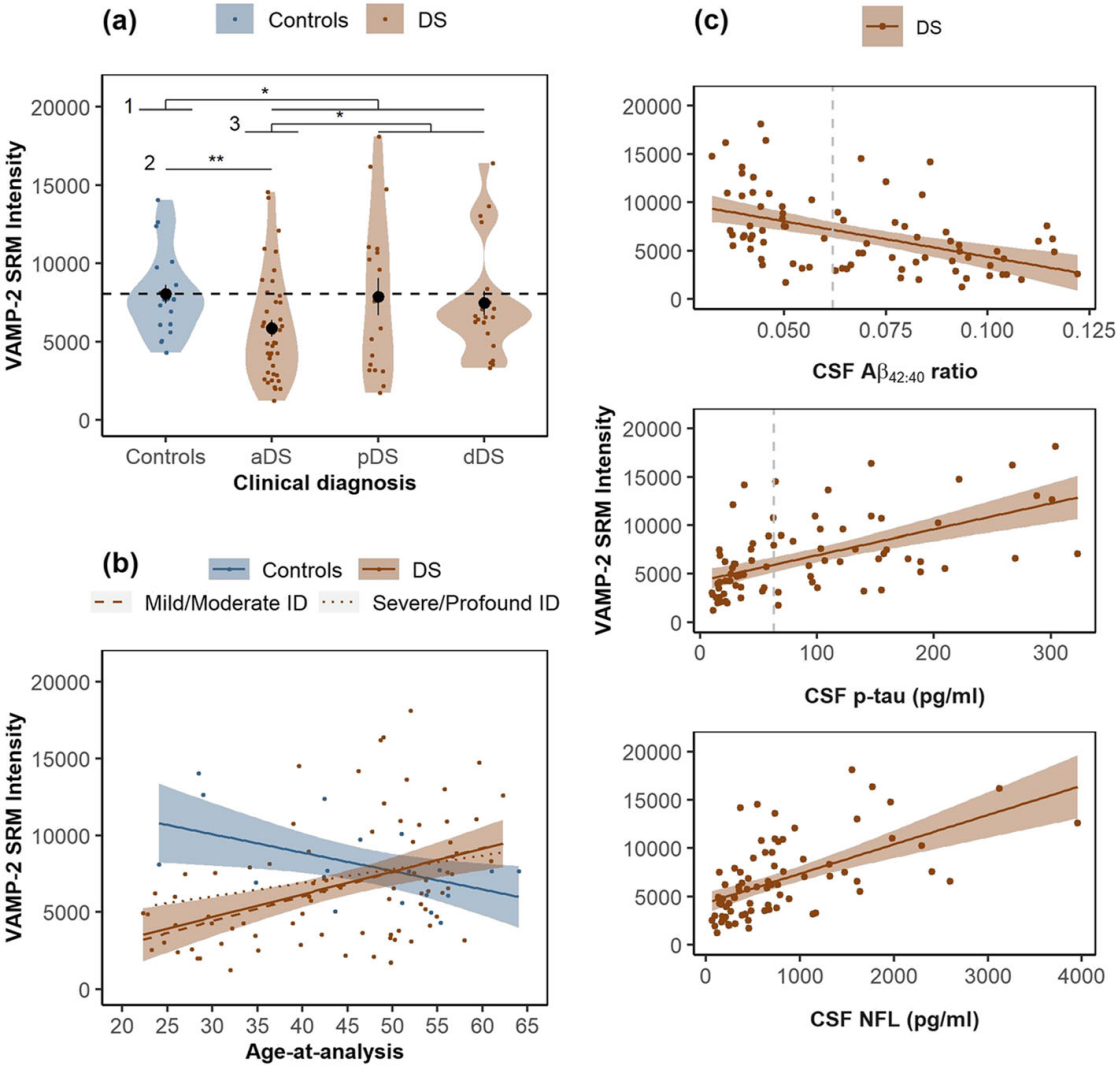
CSF levels of VAMP-2 in non-trisomic controls and DS. **a** Violin plots show the distribution of SRM intensities for VAMP-2 quantified in CSF for non-trisomic cognitively normal subjects (controls) and adults with DS across AD stages; asymptomatic AD (aDS), prodromal AD (pDS) or AD dementia (dDS). The horizontal dotted line represents the mean value in controls. *p < .05, **p < .01 for linear regression using square root transformed VAMP-2 levels in 1 DS vs controls, 2 aDS vs controls and 3 pDS/dDS vs aDS. **b** Age-at-analysis (years) is plotted against VAMP-2 SRM intensities in controls and adults with DS. **c** CSF biomarkers; Aβ_42:40_ ratio, p-tau and NFL are plotted against VAMP-2 SRM intensities in adults with DS. Linear regression lines in **b** and **c** are shown for each group (see legends). Shaded areas represent standard error of the regression lines. The vertical dotted lines in **c** represent the validated cut-offs for biomarker positivity in sporadic AD

Figure 2a shows that mean CSF VAMP-2 SRM intensities were lower in individuals with DS compared to controls (.84-fold, *p* = .04). Mean CSF VAMP-2 intensities were lower in the aDS group compared to controls (.73-fold, *p* = .01) and compared to the symptomatic group (pDS and dDS combined; .67-fold, *p* = .007). CSF VAMP-2 intensities were comparable to controls in pDS (.98-fold, *p* = .52) and dDS (.93-fold, *p* = .52). This relative increase in CSF VAMP-2 at late AD stages in adults with DS is supported by Fig. 2b, which shows that CSF VAMP-2 directly correlated with age in DS (*r* = .43, *n* = 79, *p <* .0001). This association was also observed in a linear regression analyses adjusting for degree of ID (adj.*r*^*2*^ = .16, *n* = 79, *p <* .002). Conversely, CSF VAMP-2 inversely correlated with age in controls (*r* = −.51, *n* = 20, *p* = .02). The control and DS regression lines for VAMP-2 were non-overlapping at the earliest age included in the study (22 years old) and did not intercept until the age of 42. CSF VAMP-2 was not associated with AD diagnosis when controlling for age *p* = .61).

Figure 2c shows the correlation between CSF VAMP-2 and CSF biomarkers of brain amyloid and tau pathology and axonal degeneration in adults with DS. VAMP-2 inversely correlated with the Aβ_42:40_ ratio (*r* = − .47, *n* = 79, *p <* .0001) and directly correlated with p-tau, (*r* = .56, *n* = 78, *p <* .0001) and NFL (*r* = .57, *n* = 78, *p <* .0001). To determine whether low CSF VAMP-2 is related to AD biomarker positivity in asymptomatic DS, we compared CSF VAMP-2 SRM intensities in the aDS group stratified by positivity for CSF Aβ_1-42_ using our validated in-house cut-offs for sporadic AD. Compared to controls, CSF VAMP-2 SRM intensities were lower in individuals positive for CSF Aβ_1-42_ (0.67-fold, *n* = 17, p = .009) but not in individuals negative for CSF Aβ_1-42_ (0.78-fold, *n* = 23, *p* = .30). Thus, low CSF VAMP-2 is related to AD biomarker positivity and changes over the course of AD and with age in adults with DS.

### CSF VAMP2 is associated with cognitive performance in adults with DS

**Fig. 3:**
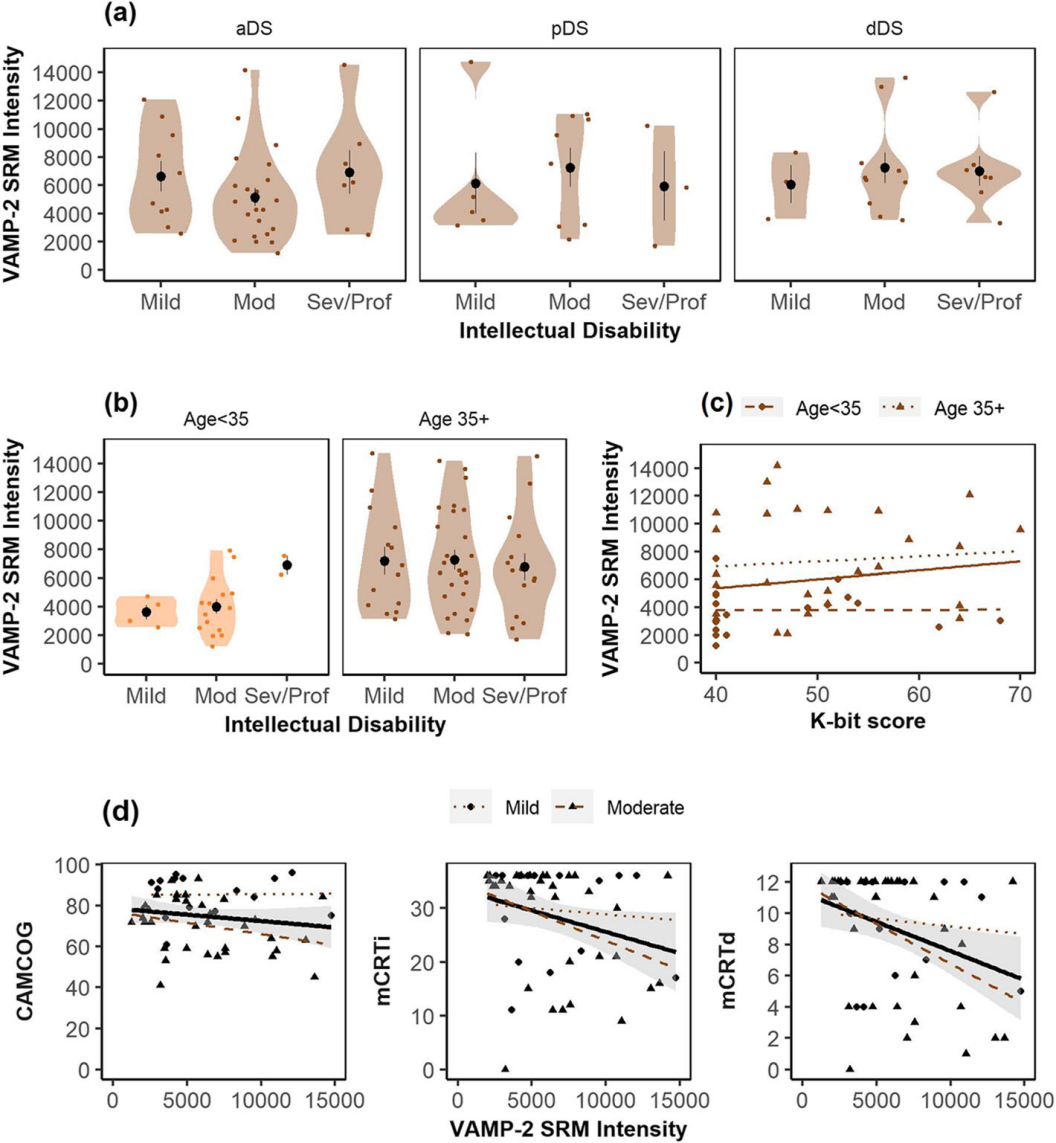
Relationship between CSF VAMP-2 and measures of intellectual impairment and cognitive performance in DS. Violin plots show the distribution of CSF VAMP-2 SRM intensities in adults with DS grouped according to degree of intellectual disability and **a** AD diagnosis and **b** age group. Circles represent mean intensities and error bars represent standard error of the mean. VAMP-2 SRM intensities in adults with DS are plotted against quantitative measures of intellectual disability (**c**) and cognitive performance (**d**). Linear regression lines are shown for models in total DS dataset and standard error of the regression lines are shown as shaded region. Regression lines for individuals aged < 35 or aged 35+ (**c**) or with mild or moderate ID (**d**) are also shown (see legends)

We next explored the relationship between CSF VAMP-2 and measures of intellectual and cognitive impairment in adults with DS. Mean CSF VAMP-2 SRM intensities were comparable across individuals with, mild, moderate, and severe intellectual disability (Fig. 3a) in aDS *p* = .31), pDS (*p* = .71), and dDS (*p* = .73). To determine whether neurodevelopmental factors may influence CSF VAMP-2 concentrations, we compared VAMP-2 across ID groups stratified by < 35 or 35+ (Fig. 3b). CSF VAMP-2 intensities were comparable across individuals with mild, moderate, or severe/profound ID in the younger (*p* = .15) and older (*p* = .87) age groups. Furthermore, CSF VAMP-2 SRM intensities were not associated with K-bit score (Fig. 3c) when analyzed independently (*r*^*2*^ = .02, *p* = .11) or when controlling for age (*r*^*2*^ = .19, *p* = .77). While Fig. 3d shows a similar regression line for VAMP-2 with CAMCOG and mCRT scores, correlation analyses showed that VAMP-2 SRM intensities were associated with immediate (*r* = − .32, *p* = .02) and delayed (*r* = − .36, *p* = .007) recall in the mCRT test but not with CAMCOG scores (*r* = − .19, *p* = .17). However, since ID had a greater impact on CAMCOG score than on mCRT in our previous study [22], we performed regression analysis of VAMP-2 and cognitive performance including level of ID as a covariate. We found that both ID (*t* = − 4.04, *p =* .0002) and VAMP-2 (*t* = − 2.05, *p* = .04) were associated with CAMCOG score (model *r*^*2*^ = .27, *p* = .00002), while VAMP-2 (*t* = − 2.61, *p* = .01) but not ID (*t* = − 0.69, *p* = .49) was associated with immediate recall in the mCRT test (model *r*^*2*^ = .10, *p = .03*). We observed a similar association with delayed recall (VAMP-2; *t* = − 2.94, *p* = .005, ID *t* = − 0.76, *p* = .45). Therefore, VAMP-2 was associated with both CAMCOG and mCRT score in adults with DS even when controlling for ID. VAMP-2 was not associated with any of the cognitive measures when age was included as a covariate (all *p* > .43).

### Compared to other synaptic proteins, VAMP-2 is the best correlate of cognitive performance, age, and CSF amyloid and neurodegeneration markers in adults with DS

**Fig. 4:**
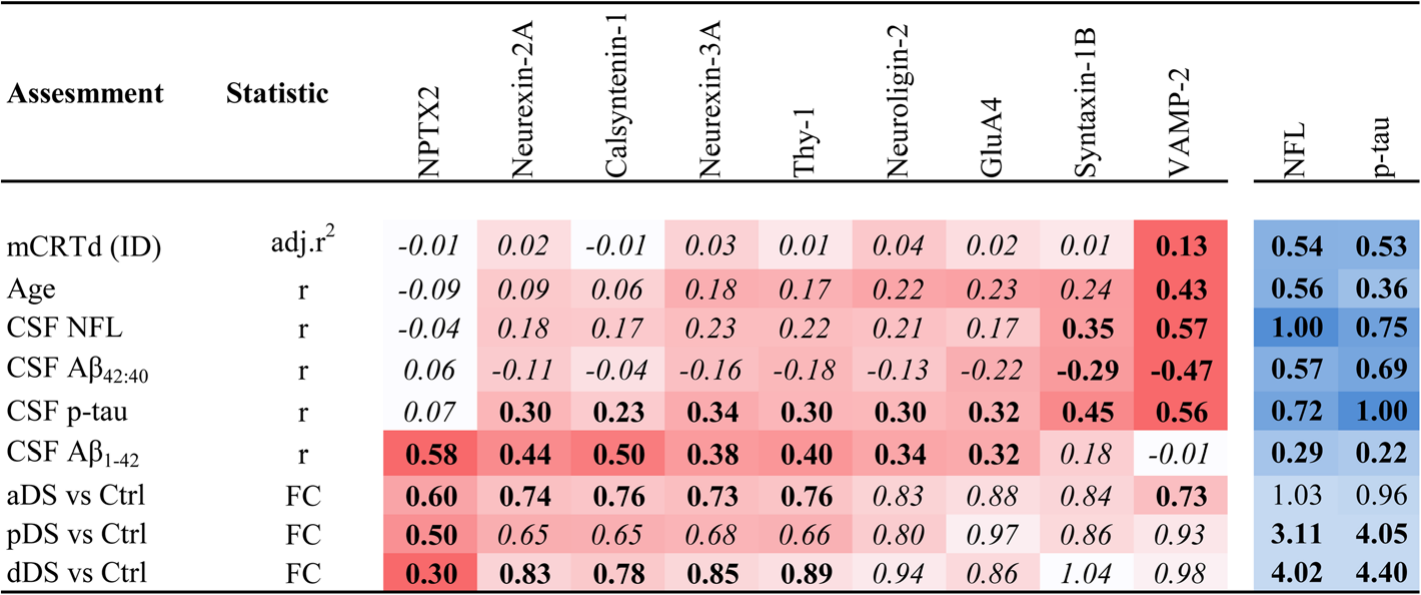
Comparison of CSF profile of 9 synaptic proteins in adults with DS. Assessment of the 9 synaptic proteins and their association (adj. r^2^ or r coefficients) with immediate and delayed recall in the mCRT test (mCRTi and mCRTd), CAMCOG, age, CSF NFL, Aβ_42:40_, p-tau and Aβ_1-42_ and the fold-change (FC) compared to controls in adults with DS across clinical groups. Associations of NFL and p-tau with the same variables are shown for comparison (red for synaptic proteins, blue for NFL and p-tau). Degree of shading is relative to r coefficients or FC. Values are shown in bold where *p* < .05 and italicized where adjusted *p* > .05 (in the case of synaptic proteins this is Benjamini-Hochberg adjusted p). Quantification of NPTX2 and GluA4 and FC for NPTX2 have been published previously in [17]

Finally, we compared these findings for VAMP-2 to the other synaptic panel proteins and to NPTX2 and applied a strict adjustment of p values to account for multiple testing. Calsyntenin-1 (*p* = .03), Neuroligin-2 (*p* = .02), Neurexin-2A (*p* = .02), Neurexin-3A (*p* = .02), and Thy-1 (*p* = .03) were associated with ID in individuals aged < 35, suggesting some influence of neurodevelopmental factors on CSF concentrations of these proteins, albeit that the associations did not survive adjustment for multiple testing (all adj.*p* < .14). The correlation of VAMP-2 with immediate recall (*adj.p* = .17) and association with CAMCOG (*adj.p* = .41) did not survive adjustment for multiple testing. Variables associated with at least one synaptic protein (*adj.p* < .05) in DS are shown in Fig. 4. The associations of CSF NFL and p-tau with each variable are also shown for comparison. VAMP-2 was the only synaptic protein to correlate with mCRT delayed recall (*adj.p* = .04) and age (*adj.p* = .0008) and was the best correlate of CSF Aβ_42:40_ (*adj.p* = .0001), CSF p-tau (*adj.p <* .0001), and CSF NFL (*adj.p <* .0001). On the other hand, NPTX2 was the best correlate of CSF Aβ_1-42_ (r = .58, *adj.p* < .0001), showed the greatest fold-change across all AD stages (0.34 to 0.55-fold, adj.*p* < .002), and was the only synaptic protein to show changes in pDS (0.47-fold, adj.*p =* .002). NFL and p-tau remain the best correlates of cognitive performance in this population and were not altered in the aDS group compared to controls (1.03 fold-change, *p* = .56 and 0.96 fold-change, *p* = .93).

## Discussion

Here, we report a comprehensive evaluation of synaptic proteins in CSF from adults with DS across the whole clinical continuum of AD. We show that of the 9 synaptic proteins evaluated, VAMP-2 is the only correlate of cognitive performance and age in this relatively understudied population. We also show that while mean CSF VAMP-2 levels were lower in asymptomatic adults with DS compared to cognitively normal controls, mean VAMP-2 levels were elevated at advanced AD stages. Increased CSF VAMP-2 correlated with low CSF Aβ_42/40,_ increased CSF p-tau and NFL and worse cognitive performance. Thus, changes in CSF VAMP-2 are closely related to CSF AD biomarkers and cognitive measures in adults with DS.

In controls, CSF VAMP-2 levels decreased with age and when compared across similar ages, VAMP-2 levels were lower in adults with DS compared to controls from the earliest age included in the study (22 years old) and did not converge until the age of 42. This finding suggests a distinct CSF profile of VAMP-2, and potentially a different mechanism of synaptic pruning, between healthy aging and the presence of AD pathology and/or triplication of chromosome 21. It is possible that the relatively lower CSF VAMP-2 levels in younger adults with DS compared to controls is a result of reduced VAMP-2 expression from birth due to neurodevelopmental factors. However, several lines of evidence suggest that CSF VAMP-2 levels change as a function of AD as opposed to ID: (a) the association between CSF VAMP-2 and age was independent of ID, (b) CSF VAMP-2 was comparable to controls in adults with DS negative for the CSF amyloid marker, (c) the findings reported here for this genetically determined form of AD replicate the nonlinear CSF profile of the 8 synaptic panel proteins previously reported across disease stages in sporadic AD [18], and (d) CSF VAMP-2 did not correlate with K-bit score and was comparable between individuals classified as having mild, moderate, severe, or profound intellectual impairment across all AD stages an in individuals aged < 35 (where AD pathology is less likely to be a confounding factor), but did correlate with age, AD biomarkers, and episodic memory performance. Based on these findings, we propose that low VAMP-2 levels in these individuals may at least partially reflect changes related to the preclinical phase of AD, similar to that previously report in sporadic AD where CSF VAMP-2 levels were nominally reduced in preclinical AD and significantly elevated in prodromal and dementia stages compared to cognitively normal controls [18].

We have previously evaluated NPTX2 as a synaptic marker in the same cohort reported here [17]. Similar to VAMP-2, CSF NPTX2 levels were lower in DS compared to controls, albeit that NPTX2 was reduced at all AD stages. In fact, here we report that compared to the other 8 synaptic proteins, NPTX2 was the only protein to be reduced at all AD stages compared to controls. However, unlike VAMP-2, CSF NPTX2 did not correlate with cognition or age. The distinct expression and function of these two proteins could explain their distinct CSF profiles in adults with DS. VAMP-2 is expressed at the human cortical synapse with increased enrichment compared not only to the other 7 synaptic panel proteins evaluated here but also to the widely used pre-synaptic marker, synaptophysin [18]. This high synapse specificity supports other studies that have shown that VAMP-2 is predominantly found at glutamatergic synapses [31] as part of the synaptic exocytosis core vesicular complex [32] where it is necessary for regulating the releasable pool of glutamate at the pre-synapse [33] and is also critical for post-synaptic trafficking of glutamate receptor subunits, particularly in the CA1 region of the hippocampus [34]. Reduced VAMP-2 brain expression has been reported in AD [35]. NPTX2 is specifically expressed by pyramidal neurons where it mediates activity-dependent strengthening of pyramidal neuron excitatory synapses on GABAergic parvalbumin interneurons [10]. Therefore, both VAMP-2 and NPTX2 are specifically expressed at distinct populations of synapses where they have distinct functions that are critical for synaptic transmission. We therefore propose that CSF levels of these 2 synaptic proteins may reflect degeneration of distinct synapse populations. While NPTX2 remains a promising surrogate marker of inhibitory circuit dysfunction in AD, DS, and other neurodegenerative diseases, VAMP-2 may be a better surrogate marker of cognitive performance in adults with DS.

A previous study reported that CSF NPTX2 correlates well CSF levels of two other synaptic proteins, SNAP-25 and neurogranin in sporadic AD CSF [12]. In this study, we report that, with the exception of VAMP-2, CSF levels of synaptic proteins were highly inter-correlated in adults with DS and that VAMP-2 was the only synaptic protein not to correlate with at least one other synaptic protein in controls.

The novelty of VAMP-2 is that it was the only synaptic protein evaluated here to correlate with age (a surrogate measure of disease progression in DS) and mCRT in the DS population. Similar to our previous study, we found that ID had a greater impact on CAMCOG score than on mCRT [22] such that the association of VAMP-2 with mCRT score was evident without the need to control for level of ID. The mCRT test is a version of the CRT modified for use in DS and discriminates well between DS adults with and without dementia [26]. The CRT test is considered a clinical marker of episodic memory disorders due to medial temporal damage, especially in the CA1 field of the hippocampus [36], which is consistent with the functional role of VAMP-2 at CA1 synapses [34].

## Study limitations

While DS and autosomal dominant cohorts with available CSF are scarce, further replication of the findings reported here in independent genetic AD and DS cohorts would be valuable. A limitation of this study is the cross-sectional design, particularly in the analysis of cognitive decline. Longitudinal studies are needed to fully establish the prognostic value of VAMP-2 in DS cohorts.

## Conclusion

NFL remains the best CSF correlate of cognition in this population and this work opens the door to future studies exploring the prognostic capacity of CSF VAMP-2 in adults with DS and sporadic AD. The data reported in this manuscript show proof-of-concept for CSF VAMP-2 as a potential marker of synapse degeneration that correlates with CSF AD and axonal degeneration markers and cognitive performance. Whether VAMP-2 could be a useful addition to NFL to specifically monitor synapse engagement and therapeutic response, particularly in AD clinical trials would be an interesting avenue worth pursuing; as anti-tau and anti-Aβ are common therapies, there is a need for an alternative surrogate measure of cognitive performance not directly affected by the drug. An ELISA-based immunoassay to facilitate the quantification of VAMP-2 in patient CSF is in development. Moreover, as VAMP-2 is also detectable in blood [37], whether plasma VAMP-2 can be used as a surrogate marker of brain VAMP-2 is also worth pursuing.

## Data Availability

The datasets used and/or analyzed during the current study are available from the corresponding author on reasonable request.

## Abbreviations

AD: Alzheimer’s disease
aDS: Asymptomatic Alzheimer’s disease in Down syndrome
CAMCOG: Cambridge Cognition Examination
CSF: Cerebrospinal fluid
DABNI: Down Alzheimer Barcelona Neuroimaging Initiative
dDS: Dementia due to Alzheimer’s disease in Down syndrome
DS: Down syndrome
DSM: Diagnostic and Statistical Manual of Mental Disorders
KBIT: Kaufmann Brief Intelligence Test
ID: Intellectual disability
mCRT: Modified cued recall test
NPTX2: Neuronal pentraxin-2
pDS: Prodromal Alzheimer’s disease in Down syndrome
SD: Standard deviation
SPIN: Sant Pau Initiative in Neurodegeneration
SRM: Selected reaction monitoring
VAMP-2: Vesicle-associated membrane protein-2

## References

[1] McCarron M, McCallion P, Reilly E, Mulryan N (2014). A prospective 14-year longitudinal follow-up of dementia in persons with Down syndrome. J Intellect Disabil Res.

[2] Sinai A, Mokrysz C, Bernal J, Bohnen I, Bonell S, Courtenay K, Dodd K, Gazizova D, Hassiotis A, Hillier R, McBrien J, McCarthy J, Mukherji K, Naeem A, Perez-Achiaga N, Rantell K, Sharma V, Thomas D, Walker Z, Whitham S, Strydom A (2018). Predictors of Age of Diagnosis and Survival of Alzheimer’s disease in Down syndrome. J Alzheimers Dis.

[3] Hithersay R, Startin CM, Hamburg S, Mok KY, Hardy J, Fisher EMC, et al. Association of dementia with mortality among adults with Down syndrome older than 35 years. JAMA Neurol. 2018. 10.1001/jamaneurol.2018.3616 PMC6439956 30452522

[4] Fortea J, Vilaplana E, Carmona-Iragui M, Benejam B, Videla L, Barroeta I, et al. Clinical and biomarker changes of Alzheimer’s disease in adults with Down Syndrome: a cross-sectional study. Lancet. (in press 2020). 10.1016/S0140-6736(20)30689-9 PMC7322523 32593336

[5] Selkoe DJ (2002). Alzheimer’s disease is a synaptic failure. Science..

[6] Terry RD, Masliah E, Salmon DP, Butters N, DeTeresa R, Hill R, Hansen LA, Katzman R (1991). Physical basis of cognitive alterations in Alzheimer’s disease: synapse loss is the major correlate of cognitive impairment. Ann Neurol.

[7] Henstridge CM, Sideris DI, Carroll E, Rotariu S, Salomonsson S, Tzioras M, McKenzie CA, Smith C, von Arnim CAF, Ludolph AC, Lulé D, Leighton D, Warner J, Cleary E, Newton J, Swingler R, Chandran S, Gillingwater TH, Abrahams S, Spires-Jones TL (2018). Synapse loss in the prefrontal cortex is associated with cognitive decline in amyotrophic lateral sclerosis. Acta Neuropathol.

[8] Koffie RM, Hyman BT, Spires-Jones TL (2011). Alzheimer’s disease: synapses gone cold. Mol Neurodegener.

[9] Robinson JL, Molina-Porcel L, Corrada MM, Raible K, Lee EB, Lee VM (2014). Perforant path synaptic loss correlates with cognitive impairment and Alzheimer’s disease in the oldest-old. Brain..

[10] Chang MC, Park JM, Pelkey KA, Grabenstatter HL, Xu D, Linden DJ, Sutula TP, McBain CJ, Worley PF (2010). Narp regulates homeostatic scaling of excitatory synapses on parvalbumin-expressing interneurons. Nat Neurosci.

[11] Xiao MF, Xu D, Craig MT, Pelkey KA, Chien CC, Shi Y, et al. NPTX2 and cognitive dysfunction in Alzheimer’s disease. Elife. 2017;6. 10.7554/eLife.23798. 10.7554/eLife.23798 PMC5404919 28440221

[12] Galasko DR, Xiao M, Xu D, Smirnov D, Salmon DP, Dewit N, Vanbrabant J, Jacobs D, Vanderstichele H, Vanmechelen E, Worley P, Alzheimer's Disease Neuroimaging Initiative (ADNI) (2019). Synaptic biomarkers in CSF aid in diagnosis, correlate with cognition and predict progression in MCI and Alzheimer’s disease. Alzheimers Dement.

[13] Swanson A, Willette AA (2016). Neuronal Pentraxin 2 predicts medial temporal atrophy and memory decline across the Alzheimer’s disease spectrum. Brain Behav Immun.

[14] Shao K, Shan S, Ru W, Ma C. Association between serum NPTX2 and cognitive function in patients with vascular dementia. Brain Behav. 2020:e01779. 10.1002/brb3.1779 PMC7559607 32748547

[15] van der Ende EL, Xiao M, Xu D, Poos JM, Panman JL, Jiskoot LC, Meeter LH, Dopper EG, Papma JM, Heller C, Convery R, Moore K, Bocchetta M, Neason M, Peakman G, Cash DM, Teunissen CE, Graff C, Synofzik M, Moreno F, Finger E, Sánchez-Valle R, Vandenberghe R, Laforce R Jr, Masellis M, Tartaglia MC, Rowe JB, Butler CR, Ducharme S, Gerhard A, Danek A, Levin J, Pijnenburg YA, Otto M, Borroni B, Tagliavini F, de Mendonca A, Santana I, Galimberti D, Seelaar H, Rohrer JD, Worley PF, van Swieten J, Genetic Frontotemporal Dementia Initiative (GENFI) (2020). Neuronal pentraxin 2: a synapse-derived CSF biomarker in genetic frontotemporal dementia. J Neurol Neurosurg Psychiatry.

[16] van Steenoven I, Koel-Simmelink MJA, Vergouw LJM, Tijms BM, Piersma SR, Pham TV, Bridel C, Ferri GL, Cocco C, Noli B, Worley PF, Xiao MF, Xu D, Oeckl P, Otto M, van der Flier WM, de Jong FJ, Jimenez CR, Lemstra AW, Teunissen CE (2020). Identification of novel cerebrospinal fluid biomarker candidates for dementia with Lewy bodies: a proteomic approach. Mol Neurodegener.

[17] Belbin O, Xiao MF, Xu D, Carmona-Iragui M, Pegueroles J, Benejam B, Videla L, Fernández S, Barroeta I, Nuñez-Llaves R, Montal V, Vilaplana E, Altuna M, Clarimón J, Alcolea D, Blesa R, Lleó A, Worley PF, Fortea J (2020). Cerebrospinal fluid profile of NPTX2 supports role of Alzheimer’s disease-related inhibitory circuit dysfunction in adults with Down syndrome. Mol Neurodegener.

[18] Lleo A, Nunez-Llaves R, Alcolea D, Chiva C, Balateu-Panos D, Colom-Cadena M (2019). Changes in synaptic proteins precede neurodegeneration markers in preclinical Alzheimer’s disease cerebrospinal fluid. Mol Cell Proteomics.

[19] Alcolea D, Clarimon J, Carmona-Iragui M, Illan-Gala I, Morenas-Rodriguez E, Barroeta I (2019). The Sant Pau Initiative on Neurodegeneration (SPIN) cohort: a data set for biomarker discovery and validation in neurodegenerative disorders. Alzheimers Dement.

[20] Fortea J, Carmona-Iragui M, Benejam B, Fernandez S, Videla L, Barroeta I (2018). Plasma and CSF biomarkers for the diagnosis of Alzheimer’s disease in adults with Down syndrome: a cross-sectional study. Lancet Neurol.

[21] Alcolea D, Pegueroles J, Munoz L, Camacho V, Lopez-Mora D, Fernandez-Leon A (2019). Agreement of amyloid PET and CSF biomarkers for Alzheimer’s disease on Lumipulse. Ann Clin Transl Neurol.

[22] Benejam B, Videla L, Vilaplana E, Barroeta I, Carmona-Iragui M, Altuna M, Valldeneu S, Fernandez S, Giménez S, Iulita F, Garzón D, Bejanin A, Bartrés-Faz D, Videla S, Alcolea D, Blesa R, Lleó A, Fortea J (2020). Diagnosis of prodromal and Alzheimer’s disease dementia in adults with Down syndrome using neuropsychological tests. Alzheimers Dement (Amst).

[23] Kaufman AS, Kaufman N (1990). L. Manual for the Kaufman Brief Intelligence Test. : Circle Pines.

[24] Esteba-Castillo S, Dalmau-Bueno A, Ribas-Vidal N, Vila-Alsina M, Novell-Alsina R, Garcia-Alba J (2013). Adaptation and validation of CAMDEX-DS (Cambridge Examination for Mental Disorders of Older People with Down’s Syndrome and others with intellectual disabilities) in Spanish population with intellectual disabilities. Rev Neurol.

[25] Devenny DA, Zimmerli EJ, Kittler P, Krinsky-McHale SJ (2002). Cued recall in early-stage dementia in adults with Down’s syndrome. J Intellect Disabil Res.

[26] Benejam B, Fortea J, Molina-Lopez R, Videla S (2015). Patterns of performance on the modified cued recall test in Spanish adults with Down syndrome with and without dementia. Am J Intellect Dev Disabil.

[27] Teunissen CE, Tumani H, Bennett JL, Berven FS, Brundin L, Comabella M (2011). Consensus guidelines for CSF and blood biobanking for CNS biomarker studies. Mult Scler Int.

[28] Alcolea D, Martinez-Lage P, Sanchez-Juan P, Olazaran J, Antunez C, Izagirre A (2015). Amyloid precursor protein metabolism and inflammation markers in preclinical Alzheimer disease. Neurology..

[29] Choi M, Chang CY, Clough T, Broudy D, Killeen T, MacLean B, Vitek O (2014). MSstats: an R package for statistical analysis of quantitative mass spectrometry-based proteomic experiments. Bioinformatics..

[30] R-Core-Team (2018). R: A language and environment for statistical computing.

[31] Benagiano V, Lorusso L, Flace P, Girolamo F, Rizzi A, Bosco L, Cagiano R, Nico B, Ribatti D, Ambrosi G (2011). VAMP-2, SNAP-25A/B and syntaxin-1 in glutamatergic and GABAergic synapses of the rat cerebellar cortex. BMC Neurosci.

[32] Lin RC, Scheller RH (1997). Structural organization of the synaptic exocytosis core complex. Neuron..

[33] Gu Y, Chiu SL, Liu B, Wu PH, Delannoy M, Lin DT, et al. Differential vesicular sorting of AMPA and GABAA receptors. Proc Natl Acad Sci U S A. 2016. 10.1073/pnas.1525726113 PMC4763724 26839408

[34] Hussain S, Davanger S (2015). Postsynaptic VAMP/synaptobrevin facilitates differential vesicle trafficking of GluA1 and GluA2 AMPA receptor subunits. PLoS One.

[35] Berchtold NC, Coleman PD, Cribbs DH, Rogers J, Gillen DL, Cotman CW (2012). Synaptic genes are extensively downregulated across multiple brain regions in normal human aging and Alzheimer’s disease. Neurobiol Aging.

[36] Sarazin M, Chauvire V, Gerardin E, Colliot O, Kinkingnehun S, de Souza LC (2010). The amnestic syndrome of hippocampal type in Alzheimer’s disease: an MRI study. J Alzheimers Dis.

[37] Uhlen M, Oksvold P, Fagerberg L, Lundberg E, Jonasson K, Forsberg M, Zwahlen M, Kampf C, Wester K, Hober S, Wernerus H, Björling L, Ponten F (2010). Towards a knowledge-based Human Protein Atlas. Nat Biotechnol.

